# Secondary prevention using a statin with or without aspirin among Ghanaian intracerebral hemorrhage survivors: A feasibility randomized trial

**DOI:** 10.1016/j.neuros.2025.100008

**Published:** 2025-09

**Authors:** Fred Stephen Sarfo, Hanson Ababio, Priscilla A Opare-Addo, Vida Obese, Manolo Agbenorku, Rexford Adu-Gyamfi, Sheila Adamu, John Akassi, Samuel Blay Nguah, Bruce Ovbiagele

**Affiliations:** a Komfo Anokye Teaching Hospital, Kumasi, Ghana; b Kwame Nkrumah University of Science and Technology, Kumasi, Ghana; c Kwame Nkrumah University of Science and Technology Hospital, Kumasi, Ghana; d Agogo Presbyterian Hospital, Agogo, Ghana; e University of California San Francisco, San Francisco, USA

**Keywords:** Intracerebral hemorrhage, recurrence, Safety, Africans, Prevention

## Abstract

**Background:**

Intracerebral hemorrhage (ICH) survivors are at high risk for future ischemic vascular events and recurrent ICH. Due to an uncertain risk-benefit ratio, practice guidelines equivocate on whether antiplatelet therapies or statins should be prescribed for secondary prevention in the aftermath of a spontaneous ICH.

**Purpose:**

To preliminarily assess the tolerability and safety of low dose aspirin with or without atorvastatin at low and high doses for secondary prevention after ICH.

**Methods:**

In this single-center feasibility trial in Ghana, participants were randomized to 1 of 6 arms: no aspirin (ASA-0) + no atorvastatin (ATO-0) *n* = 10); aspirin 75 mg + no atorvastatin (ASA-75 + ATO-0, *n* = 10); no aspirin + atorvastatin 20 mg (ASA-0 + ATO-20, *n* = 10); no aspirin + atorvastatin 80 mg (ASA-0 + ATO-80, *n* = 10); aspirin 75 mg + atorvastatin 20 mg (ASA-75 + ATORVA-20, *n* = 11); and aspirin 75 mg + atorvastatin 80 mg (ASA-75 + ATORVA 80, *n* = 11) taken daily for 12 months. All participants received standard of care secondary prevention comprising of antihypertensive medications for blood pressure control. Outcomes assessed included major adverse cardiovascular events (stroke, myocardial infarction, vascular death), treatment-limiting side effects, change in Modified Rankin score and global cognition.

**Results:**

We enrolled 62 eligible recent ICH survivors between January 24, 2021, and March 16, 2022, mean (SD) age of 53 (11.6) years, 30 (48 %) were males. There were 3 deaths, one in each of these arms: ASA-0 + ATO-0; ASA-0 + ATO-20; ASA-0 + ATO-80. There was one non-fatal, ischemic stroke in the ASA-75 + ATO-80 arm and one non-fatal ICH in the ASA-75 + ATO-20 arm. No treatment limiting side effects were recorded.

**Conclusion:**

These feasibility and preliminary data suggest that aspirin with or without atorvastatin up to one year after a spontaneous ICH is well tolerated and may not raise recurrent ICH risk. A definitive large study with longer term follow-up is warranted.

## Introduction

The 2022 American Heart Association guidelines on secondary prevention after surviving spontaneous intracerebral hemorrhage (ICH) do not provide definitive recommendations on the use of antithrombotic and statins.^1^ Evidence from Get With The Guidelines-Stroke registry in the US shows that 10.4 % and 35.1 % of a cohort of approximately 500,000 ICH survivors between 2011 and 2021 were prescribed antithrombotic and statins respectively.^2^ Survivors of ICH are at a 2- to 3-fold higher risk of developing major cardiovascular events, notably ischemic stroke, myocardial infarction, and vascular death leading to adverse long-term outcomes.^3–6^ Importantly, ICH survivors share atherosclerotic risk factors with ischemic stroke, myocardial infarction, and peripheral vascular disease. Among Ghanaian ICH survivors, up to 65 % had Framingham Risk Score in the intermediate and high-risk categories.^7^ Furthermore, in sub-Saharan Africa the proportion of stroke due to ICH is almost 30 % compared with 10 % in high-income countries, with ICH affecting a younger age group with a mean age of 50 years on the African continent.

There is clinical and epidemiological equipoise on the safety and benefit of anti-atherosclerotic agents after ICH. Regarding statins, the SPARCL trial (Stroke Prevention by Aggressive Reduction in Cholesterol Levels)^8^ and the HPS (Heart Protection Study)^9^ have suggested that individuals with stroke, particularly those with ICH, are at heightened risk of subsequent ICH. However, meta-analyses of high-quality observational studies ^10,11^, including a population-based, propensity score-matched Danish cohort with 2728 ICH and 52,964 ischemic stroke survivors matched for statin and non-statin use with up to 10 years follow-up found no evidence that statins increased risk of ICH.^12^ Concerning Aspirin, the RESTART trial suggested that resumption of antiplatelets after ICH was safe.^13^ A meta-analysis of 6 cohort studies involving 1916 patients found that resumption of aspirin was associated with a decreased risk of ischemic or thromboembolic events (relative risk, 0.61; 95 % CI: 0.48–0.79) with no increased risk of ICH recurrence (relative risk, 0.84; 95 %CI: 0.47–1.51).^14^ These emerging lines of evidence which were published after the 2022 AHA guidelines seem to suggest that statins or antiplatelets use after ICH may be safe depending on patient comorbidities.

This critical hiatus in the clinical management of ICH survivors requires systematic and dedicated studies to provide answers for patients, their relatives, clinicians, and policy makers. Questions around the timing of use of anti-atherosclerotic agents, dosages and whether these agents can be combined in ICH patients as is done for ischemic stroke survivors are unanswered. In the present feasibility trial, we aimed to derive estimates of incidence of major adverse cardiovascular events and safety outcomes for secondary preventive strategies aimed at atherosclerotic risk reduction after surviving an ICH. We compared standard of care secondary prevention after ICH (no aspirin and no atorvastatin) with low dose aspirin with or without atorvastatin at low and high doses for secondary prevention after ICH.

## Methods

Study design and participants: This was a feasibility, six-arm, parallel, open-label, assessor blinded, randomized clinical trial among recent survivors with CT scan confirmed intracerebral hemorrhage. Institutional approval for the study was obtained from the Committee of Human Research Publication and Ethics (CHRPE/AP/821/20) in Kumasi, Ghana. Kumasi is a metropolitan city with a population of 4 million people and the neurology unit of KATH which admits approximately 900 people with stroke annually. All patients provided written informed consent before screening for eligibility into the trial.

Eligible participants were 18 years or older, male, or female, with a recent CT scan-confirmed intracerebral hemorrhage that has occurred within 1 month before study enrollment. Participants could have the following additional conditions: documented hypertension (>140/90 mm Hg) or previous treatment with antihypertensive medications; documented diabetes or previous treatment with an oral hypoglycemic or insulin.

Exclusion criteria were the inability to sign informed consent; contraindications to Aspirin or atorvastatin; medication (such as anticoagulation) associated ICH; severe cognitive impairment, dementia; severe renal disease, renal dialysis, awaiting a renal transplant, or having received a renal transplant; cancer diagnosis or treatment in pervious 2 years; clinically significant arrhythmias (including unresolved ventricular arrhythmias or atrial fibrillation); and people who were breastfeeding or pregnant.

### Randomization and masking:

The potential study participants or reliable family proxies provided written informed consent before screening, and those meeting eligibility criteria were enrolled as in-patients prior to their discharge home from hospital after surviving an acute ICH or presenting for their first clinic review after surviving an ICH. Eligible participants were randomly allocated to one of six arms:

1. standard of care (SC)- no aspirin, no atorvastatin [ASA-0, + ATO-0];
2. SC plus aspirin 75 mg, no atorvastatin [ASA-75 + ATO-0];
3. SC plus no aspirin, low intensity atorvastatin 20 mg [ASA-0 + ATO-20];
4. SC plus aspirin 75 mg and low intensity atorvastatin 20 mg [ASA-75 + ATO-20];
5. SC plus no aspirin, high intensity atorvastatin 80 mg [ASA-0 + ATO-80]; and
6. SC plus aspirin 75 mg and high intensity atorvastatin 80 mg [ASA-75 + ATO-80] daily.
7. Soluble aspirin was taken daily for 12 months while atorvastatin at assigned doses was taken once daily.

Allocation was done by simple randomization using a computer-generated randomization sequence by study statistician. We choose a simple randomization for this feasibility trial based on its simplicity and unbiasedness in affording each participant an equal chance of being assigned into any of the six arms but mindful of the risk for imbalance between groups with this approach. Each sequence generated was concealed in an opaque brown envelope and opened by the Research Coordinator in the presence of the consenting eligible participant at enrollment. Neither participants nor investigators were masked to group assignment in this open-label pilot trial. A team of 3 experienced doctors who was not part of the study investigative team adjudicated major adverse cardiovascular events (MACE). Functional status using the Modified Ranking scale^15^ was assessed by a medical research assistant blinded to study arm allocation, who also collected other exploratory outcome measures detailed in the outcomes sub-section below.

### Procedures:

All study participants received standard of care (SC) secondary prevention for hypertensive ICH which focuses primarily on control of blood pressure using locally available antihypertensive medications with a BP control goal of 140 / 90 mm Hg. Study physicians could choose from any of the following antihypertensive drug classes (angiotensin-converting enzyme [ACE] inhibitors, angiotensin receptor blockers, beta blockers, calcium channel blockers, diuretics, and centrally acting agents) used in combination and at doses titrated to achieve BP goals. Participants in the control arm were assigned to SC while the 5 other arms received aspirin and or atorvastatin on top of standard of care.

Stroke severity was assessed at enrollment and follow-up using the modified National Institutes of Health Stroke Scale^16^ and functional status assessed using the Modified Rankin Scale (MRS) with scores from 0 (no functional limitation) to 6 (death). An assessment of vascular risk factors was performed using history and physical examination to identify the presence of hypertension, diabetes, cigarette smoking, and alcohol use. Before enrollment, participants were evaluated by study physicians to confirm the absence of contraindications to the study medications.

Participants were followed up for 12 months at our out-patient neurology clinic with scheduled visits on months 1, 3, 6, 9, and 12 to be reviewed by Senior registrars in Neurology. Adverse events were assessed via both self-reports or reports by family caregivers. Study doctors assessed adverse events and graded them as mild, moderate, severe, or serious based on the common terminology criteria for adverse events.

### Outcomes:

Outcomes for this feasibility trial were exploratory and aimed at estimating incidence of clinical events to inform the design of a future definitive trial:

1. Incidence of major adverse cardiovascular events including non-fatal strokes (ischemic or ICH), myocardial infarction, and cardio-cerebrovascular deaths.
2. Change in functional status defined as change in the modified Rankin Scale (MRS) score at month 12 from baseline score. We also defined a favorable functional outcome as an MRS score of ≤ 2 at month 12 and a score of ≥3 as unfavorable.
3. Change in cognitive performance defined as change in the Montreal Cognitive Assessment (MOCA)^17^ at month 12 from month 3. Baseline MOCA scores were not utilized to avoid the risk of post-stroke delirium which is rife within the first three months post-stroke.
4. Treatment-limiting side effects of Aspirin namely severe bleeding into major organs (brain and gastrointestinal tract) and atorvastatin namely rhabdomyolysis, and severe myalgia.
5. Treatment satisfaction score at month 12
6. Changes in Hamilton Depression Scale^18^ scores at month 12 from baseline.
7. Serious adverse events were reported to the local Institutional Review Board.

### Sample size:

No power calculations were performed. We planned to enroll 12 eligible participants per arm (a total sample size of 72) within 12 months of study initiation with 12 months of follow-up. This sample size was based not on effect size estimates but on pragmatic and feasibility considerations for a non-funded study. As suggested by Mead^19^, we sought to obtain a reasonable precision for variance estimates in preparation for future study design.

### Statistical analysis:

We compared means and medians between treatment arms using either Analysis of variance (ANOVA) test or Kruskal Walis rank sum test for continuous variables and chi-squared or Fisher’s exact tests for categorical variables to assess how well balanced the arms were at enrollment. To estimate the direction and magnitude of change in functional status and cognitive performance, we used a linear regression modelling to compare the effect of each of the 5 treatment arms with the control arm (ASA 0, ATORVA 0) with adjustment for baseline MRS and MOCA scores respectively. In these analyses, the beta coefficient and 95 %CI were reported with no adjustment for multiple testing. We also reported proportions with favorable MRS score of 2 or less at month 12 for each study arm and computed odds ratios with 95 % CIs with the control arm as referent. Multiple imputation by chain equations (MICE) was used to handle missing data. In all analyses, p-value of < 0.05 was set as the level of statistical significance. All analyses were performed with the use of R Statistical Software version 4.5.0.

## Results

### Enrollment and disposition of the study participants:

**Fig. 1.**
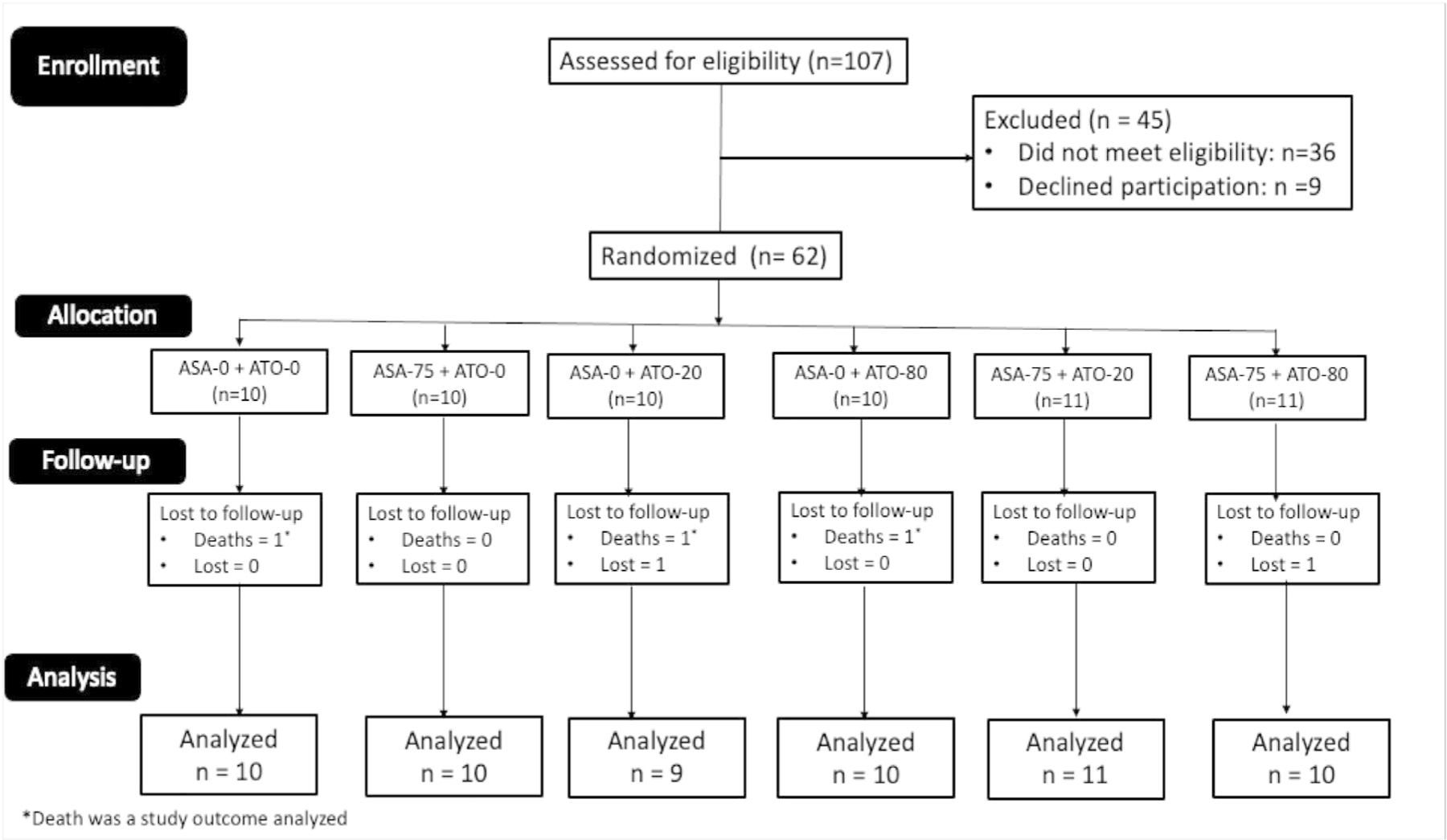
CONSORT DIAGRAM.

We were able to enroll 62 out of 72 (86 %) of the projected sample size of the study over a 15-month period. The first participant was enrolled on January 24, 2021, and the last participant March 16, 2022. The median (range) duration in days between ICH onset and enrollment was 12 (6 – 25) days. The last participant to complete follow-up was March 28, 2023. Two participants were lost to follow-up and did not complete the study, one was assigned to ASA-75+ ATO-80 and the other on ASA-0 + ATO 20. (Fig. 1)

### Baseline Characteristics of Study Participants:

We enrolled and allocated 62 eligible participants into the 6 arms of the study. The mean (SD) age of participants was 53 (11.6) years, and 30 (48 %) were males. Ten (10) participants each were randomized to [ASA-0 + ATO- 0], [ASA-75 + ATO-0], [ASA-0 + ATO-20], and [ASA-0 + ATO-80] respectively; while 11 participants each were assigned to [ASA-75 + ATO-20] and [ASA-75 + ATO-80] respectively. The participants were balanced in all key demographic and clinical characteristics except for cigarette smoking history and use of diuretics prescribed at enrollment which differed significantly across the group (Table 1). All participants had hypertension associated ICH.

### Major Clinical Events:

There were three (3) deaths (4.8 %) during follow up with one death each in the [ASA-0 + ATO-0]; [ASA-0 + ATO-20]; and [ASA-0 + ATO-80] arms respectively. All deaths occurred at home and verbal postmortem from relatives pointed to possible vascular deaths. Regarding recurrent strokes, there were two non-fatal events, one each in the [ASA- 75 + ATO-20] and [ASA-75 + ATO-80] arms. One recurrent stroke was recurrent intracerebral hemorrhage which occurred in the [ASA-75 + ATO-20] arm and the other was a lacunar infarct occurring in the [ASA-75 + ATO-80] arm. Both participants with recurrent stroke survived and were alive at month 12 of follow-up. No major clinical event was recorded in the ASA-75 + ATO-0 arm. None of these major clinical events differed significantly by study arm.

### Treatment limiting side effects of aspirin or atorvastatin:

No study participant had aspirin or atorvastatin withdrawn due to side effects.

### Functional outcomes:

A change in mean (SD) MRS score by study arm at month 12 from baseline was 0 (1.1) units for [ASA-0 + ATO-0]; 0 (0.9) units for [ASA-0 + ATO-20] arm while all other arms had at least 1 or more units improvement in the MRS score. (Table 2) Among participants who completed follow-up at month 12, 7/10 (70 %) in the [ASA-0 + ATO-0] arm; 7/10 (70 %) in the [ASA-0 + ATO-80] arm; 7/9 (78 %) in the [ASA-0 + ATO-20] arm; 8/10 (80 %) in the [ASA-75 + ATO-0] arm; 9 / 11 (81.8 %) in the [ASA-75 + ATO-20] arm; and 9/10 (90 %) in the [ASA-75 + ATO-80] arm had favorable functional outcomes defined by an MRS of 2 or lower. Upon adjustment for baseline MRS and comparing with the control [ASA- 0 + ATO-0] arm as referent, the [ASA-75 + ATO-80] group improved the most by −0.5 units (95 % CI: −1.3, 0.34) (lower is better); followed by [ASA-75 + ATO-0] arm by −0.40 units (95 % CI: −1.2, 0.44) units while the [ASA-0 + ATO-20] arm improved the least by −0.04 units (95 % CI: −0.90, 0.81) (Table 3).

### Cognitive outcomes:

A change in mean (SD) MOCA score by study arm at month 12 from month 3 was +3 (2.7) units for standard of care [ASA-0 + ATO-0], +3 (2.8) units for [ASA-0+ATO-20] arm while all other arms had at least 4 units or more improvement in MOCA score. (Table 2) Results of unadjusted and adjusted estimates of change in cognitive performance on the MOCA scale is shown in Table 4. Comparing with the control arm [ASA-0 + ATO-0] as referent, the [ASA-75 + ATO-0] group improved the most by 4.3 units followed by [ASA-75 + ATO-80] by 2.7 units while the [ASA-0 + ATO-20] declined by −0.86 units compared with the control arm with attenuation of effect sizes upon adjustment for baseline MOCA scores (Table 4).

## Discussion

In this feasibility trial we have preliminarily assessed the safety, tolerability, and signal of clinical effect of low dose aspirin with or without atorvastatin at varying doses aimed at reducing atherosclerotic risk among recent ICH survivors. The mean age of study participants was 53 years, reflecting the relatively younger age of onset of ICH in our population. We found that the overall incidence of major adverse cardiovascular events namely recurrent strokes and cardio/cerebrovascular deaths at 8.6 % over 12 months was comparable with published data. A large non-interventional Danish study among 2289 patients aged 50 years or older who had a first-ever ICH with a mean follow-up of 2.1 years reported a MACE incidence rate of 10.8 % among patients with lobar ICH, vs 7.9 % among those with deep ICH.^20^

Aspirin at fixed dosage of 75 mg with or without atorvastatin at 20 mg (low intensity) or at 80 mg (high intensity) were started within 1 to 4 weeks after an ICH event. Given the evidence suggesting that the occurrence of MACE is highest within the first 30 days after an ICH^20^, this time point chosen for initiation of anti-atherosclerotic therapy after ICH in the present study is justifiable. While the 2 recurrent strokes which occurred were observed in the arms on aspirin 75 mg plus atorvastatin 20 mg or 80 mg respectively, the 3 deaths which occurred were in the control arm, and the two arms on either atorvastatin 20 mg or 80 mg without aspirin. Furthermore, the two participants who experienced recurrent strokes had non-fatal events and both were continued on their allocated treatment and completed month 12 follow-up. There was no clinical event in the low dose aspirin only arm. Overall, the major adverse clinical events were evenly distributed over the 6 arms of the study.

The treatment regimens tested in this experimental study were well tolerated with no treatment related side effects leading to withdrawal of these medications. As depicted in Tables 3 and 4, the effect sizes of functional and cognitive outcomes by treatment arms compared with the control arm were overall modest and non-significant. The group on aspirin 75 mg plus atorvastatin 80 mg showed a relatively moderate but non-significant improvement in functional status with an adjusted beta (95 % CI) estimate of −0.50 (−1.30 to 0.34), *p* = 0.24 compared with the control arm. Furthermore, those on aspirin 75 mg without statin had a marginally nominal improvement in cognitive performance compared with control arm with an unadjusted beta estimate of 4.30 (−0.48 to 9.00), *p* = 0.08 with further attenuation of effect size in adjusted analysis. These observations were inconclusive potentially due to the small sample sizes in the groups. We acknowledge that post-stroke functional disposition and cognitive performance may be a function of several demographic and clinical characteristics of participants as well as other post-stroke interventions including rehabilitation.

There is a perception among clinicians that use of antiplatelets and statin medications after ICH may increase risk of risk of recurrent ICH. In support of this, data from both the U.S. and Ghanaian registries report low rates of utilization of these potentially beneficial interventions which could mitigate the excess risk of ischemic events after ICH.^2,7^ Guidelines do equivocate on whether to prescribe these agents which could mitigate ischemic events risk after ICH.^1^ In addition to optimal blood pressure control, it is estimated that the combination of statin and antiplatelet medications among ICH survivors could lower the risk of arterial ischemic events by 30 %.^22,23^ We did not observe an inordinately heightened risk of recurrent ICH in any of the study arms, although admittedly our sample size per group was too small to draw strong conclusions. Our study provides an impetus for further research endeavor towards establishing the longer-term efficacy and safety of anti-atherosclerotic medications which are currently used cautiously by clinicians for ICH survivors. This is an important clinical conundrum particularly in low-and-middle income settings in sub-Saharan Africa where ICH comprises 30 % of all stroke admissions and occurs at a mean age of 55 years.^21^ There are 8 on-going clinical trials on antithrombotic agents after ICH, none includes African centers, 7 RCTs focuses on use of anticoagulants in ICH survivors with atrial fibrillation. (NCT03996772, NCT03950076, NCT02565693, NCT03186729, NCT03243175, NCT03153150)^24–30^ and 1 RCT (NCT04522102)^31^, comparing starting or avoiding aspirin after ICH akin to RESTART. There is also an on-going study titled Statin Use in Intracerebral Hemorrhage Patients (SATURN) trial which is enrolling 1456 patients with lobar ICH on statins at stroke onset from the US, Canada, and Spain to assess whether continuation of statins (control) versus discontinuation (intervention) of the same statin impacts on recurrent symptomatic ICH.^32^

### Limitations:

This study was conducted in a single center with a limited sample size and thus the results are neither generalizable nor powered to provide conclusive evidence on use of aspirin and or atorvastatin after ICH. This study was not funded, hence laboratory monitoring including liver and kidney chemistries, complete blood counts, coagulation screens, neurovascular imaging studies were not done. Study medications namely aspirin and atorvastatin were obtained from generic medications assessable by the Ghanaian National Health Insurance Scheme. We could not achieve our target sample size of 12 eligible subjects per study arm over the proposed duration. Overall, this study was primarily oriented towards generating a hypothesis for future studies.

### Future directions:

To isolate the individual and combined effects of aspirin and atorvastatin in mitigating the ischemic event risk of ICH survivors, future studies should consider evaluating in a 4-arm parallel RCT design the effects of aspirin 75 mg vs atorvastatin 80 mg vs aspirin 75 mg plus atorvastatin 80 mg vs control arm (no aspirin, no atorvastatin).

## Conclusions

In this feasibility trial, we preliminarily demonstrate that aspirin with or without atorvastatin is well tolerated and did not appear to place patients at a substantially heightened risk of recurrent ICH. Larger studies with long-term follow-up are warranted.

## Figures and Tables

**TABLE 1:** Comparison of baseline demographic and clinical characteristics of Study Participants.

Characteristic	Statistic	Overall *N* = 62	Aspirin 0 + Atorvastatin 0 *N* = 10	Aspirin 75 mg + Atorvastatin 0 *N* = 10	Aspirin 0 + Atorvastatin 20mg *N* = 10	Aspirin 0 + Atorvastatin 80 mg *N* = 10	Aspirin 75 mg + Atorvastatin 20 mg *N* = 11	Aspirin 75 mg + Atorvastatin 80 mg *N* = 11
Age (years)	Mean (SD)	53 (11.6)	50 (15.1)	50 (7.2)	50 (12.9)	60 (7.5)	56 (13.0)	52 (10.7)
Sex, male ( %)	n ( %)	30 (48.4)	3 (30.0)	4 (40.0)	5 (50.0)	5 (50.0)	5 (45.5)	8 (72.7)
Educational attainment								
None	n ( %)	7 (11.5)	2 (20.0)	1 (11.1)	1 (10.0)	2 (20.0)	0 (0.0)	1 (9.1)
Primary	n ( %)	33 (54.1)	4 (40.0)	3 (33.3)	7 (70.0)	6 (60.0)	6 (54.5)	7 (63.6)
Secondary	n ( %)	18 (29.5)	4 (40.0)	3 (33.3)	2 (20.0)	2 (20.0)	5 (45.5)	2 (18.2)
Tertiary	n ( %)	3 (4.9)	0 (0.0)	2 (22.2)	0 (0.0)	0 (0.0)	0 (0.0)	1 (9.1)
Missing	N	1	0	1	0	0	0	0
Income (USD)								
0–100	n ( %)	7 (12.5)	1 (11.1)	1 (10.0)	2 (28.6)	1 (10.0)	0 (0.0)	2 (20.0)
101–250	n ( %)	33 (58.9)	4 (44.4)	4 (40.0)	3 (42.9)	6 (60.0)	9 (90.0)	7 (70.0)
251 or more	n ( %)	16 (28.6)	4 (44.4)	5 (50.0)	2 (28.6)	3 (30.0)	1 (10.0)	1 (10.0)
Missing	n	6	1	0	3	0	1	1
Type of domicile								
Rural	n ( %)	7 (11.3)	1 (10.0)	1 (10.0)	2 (20.0)	1 (10.0)	2 (18.2)	0 (0.0)
Urban	n ( %)	55 (88.7)	9 (90.0)	9 (90.0)	8 (80.0)	9 (90.0)	9 (81.8)	11 (100.0)
Cigarette smoking^#^	n ( %)	5 (8.1)	0 (0.0)	0 (0.0)	0 (0.0)	3 (30.0)	0 (0.0)	2 (18.2)
Alcohol use	n ( %)	14 (22.6)	3 (30.0)	2 (20.0)	1 (10.0)	4 (40.0)	1 (9.1)	3 (27.3)
Antihypertensive before ICH	n ( %)	35 (56.5)	4 (40.0)	7 (70.0)	5 (50.0)	5 (50.0)	7 (63.6)	7 (63.6)
Anti-platelet before ICH	n ( %)	1 (1.6)	0 (0.0)	1 (10.0)	0 (0.0)	0 (0.0)	0 (0.0)	0 (0.0)
Anti-coagulant before ICH	n ( %)	0 (0.0)	0 (0.0)	0 (0.0)	0 (0.0)	0 (0.0)	0 (0.0)	0 (0.0)
Anti-glycemics before ICH	n ( %)	2 (3.2)	1 (10.0)	0 (0.0)	0 (0.0)	1 (10.0)	0 (0.0)	0 (0.0)
Statins before ICH	n ( %)	0 (0.0)	0 (0.0)	0 (0.0)	0 (0.0)	0 (0.0)	0 (0.0)	0 (0.0)
Antihypertensive after ICH	n ( %)	59 (95.2)	10 (100.0)	10 (100.0)	8 (80.0)	9 (90.0)	11 (100.0)	11 (100.0)
Anti-diabetics after ICH	n ( %)	9 (14.5)	1 (10.0)	1 (10.0)	0 (0.0)	2 (20.0)	3 (27.3)	2 (18.2)
Calcium channel blockers	n ( %)	47 (75.8)	8 (80.0)	8 (80.0)	7 (70.0)	8 (80.0)	7 (63.6)	9 (81.8)
ARB/ACE-I	n ( %)	49 (79.0)	10 (100.0)	7 (70.0)	7 (70.0)	7 (70.0)	7 (63.6)	11 (100.0)
Diuretics^##^	n ( %)	28 (45.2)	6 (60.0)	8 (80.0)	5 (50.0)	4 (40.0)	1 (9.1)	4 (36.4)
Beta blockers	n ( %)	25 (40.3)	5 (50.0)	5 (50.0)	3 (30.0)	4 (40.0)	3 (27.3)	5 (45.5)
Hydralazine	n ( %)	16 (25.8)	4 (40.0)	1 (10.0)	3 (30.0)	1 (10.0)	3 (27.3)	4 (36.4)
Spironolactone	n ( %)	5 (8.1)	1 (10.0)	1 (10.0)	0 (0.0)	1 (10.0)	2 (18.2)	0 (0.0)
Methyldopa	n ( %)	7 (11.3)	1 (10.0)	1 (10.0)	1 (10.0)	1 (10.0)	2 (18.2)	1 (9.1)
Body mass index (kg/m^2^)	Mean (SD)	26 (11.4)	26 (5.8)	25 (3.4)	24 (4.3)	25 (7.5)	24 (5.7)	31 (24.9)
Waist-to-hip ratio	Mean (SD)	1 (0.1)	1 (0.1)	1 (0.1)	1 (0.1)	1 (0.1)	1 (0.1)	1 (0.1)
Systolic Blood pressure	Mean (SD)	157 (24.2)	151 (16.0)	154 (22.7)	154 (32.6)	165 (32.2)	157 (22.0)	163 (18.5)
Diastolic Blood pressure	Mean (SD)	98 (17.6)	98 (12.2)	97 (15.5)	98 (19.0)	103 (27.9)	95 (18.4)	96 (11.6)
No. of Antihypertensive meds	Median (Min,Max)	3 (0,5)	4 (2,5)	3 (2,5)	3 (0,5)	3 (0,5)	2 (1,4)	3 (2,4)
Random blood glucose, mmol/L	Mean (SD)	7 (2.0)	6 (2.2)	7 (2.7)	6 (1.1)	6 (1.6)	7 (2.4)	7 (1.7)
Missing	n ( %)	1	1	0	0	0	0	0
Montreal cognitive assessment score	Mean (SD)	16 (6.7)	18 (9.3)	15 (6.3)	15 (7.5)	16 (6.8)	17 (6.0)	16 (5.2)
Modified Rankin Score	Mean (SD)	2 (1.1)	2 (1.4)	2 (1.1)	2 (1.2)	3 (1.2)	2 (0.9)	2 (0.9)
National Institute of Health Stroke scale	Mean (SD)	3 (4.9)	2 (3.0)	3 (5.2)	4 (9.4)	3 (3.1)	2 (3.3)	2 (3.4)

ARB/ACE-*I* = Angiotensin receptor blocker/ angiotensin converting enzyme inhibitor.

# p-value for Fisher’s exact test = 0.030.

## p-value for Fisher’s exact test = 0.029.

**TABLE 2:** Comparison of Study Outcomes by Study Arms.

Outcome	Aspirin 0 + Atorvastatin 0 *N* = 10^1^	Aspirin 75 mg + Atorvastatin 0 *N* = 10^1^	Aspirin 0 + Atorvastatin 20mg *N* = 10^1^	Aspirin 0 + Atorvastatin 80 mg *N* = 10	Aspirin 75 mg + Atorvastatin 20 mg *N* = 11	Aspirin 75 mg + Atorvastatin 80 mg *N* = 11	p-value^2^
All-cause deaths	1	0	1	1	0	0	0.523
Recurrent strokes	0	0	0	0	1	1	>0.999
Serious adverse events	1	0	1	1	1	1	>0.999
Completion of 12 months follow-up	9	10	8	9	11	10	0.610
Treatment-limiting side effects	0	0	0	0	0	0	>0.999
Treatment satisfaction score, mean (SD)	53 (0.0)	53 (2.0)	53 (1.1)	53 (1.0)	53 (0.0)	53 (0.0)	0.777
Unknown	1	0	2	1	0	1	
Change in Modified Rankin Score from baseline	0 (1.1)	−1 (0.8)	0 (0.9)	−1 (1.2)	−1 (0.7)	−1 (0.9)	0.877
Unknown	0	0	1	0	0	1	
Montreal Cognitive Assessment, Month 12 score - month 3 score	3 (2.7)	4 (3.6)	3 (2.8)	4 (4.1)	4 (4.9)	4 (2.3)	0.970
Unknown	1	2	4	1	0	3	
Systolic Blood Pressure, Month 12 – Month 0, mean (SD), mm Hg	−12 (18.3)	−13 (23.3)	−20 (30.4)	−12 (16.1)	−18 (19.7)	−9 (16.5)	0.894
Unknown	1	0	2	1	0	1	
Diastolic Blood Pressure, Month 12 – Month 0, mean (SD), mm Hg	−10 (13.3)	−10 (15.1)	−15 (20.7)	−11 (17.0)	−8 (11.7)	2 (17.3)	0.664
Unknown	1	0	2	0	1	1	
Blood pressure at month 12 < 140 /90 mm Hg, n	6	6	7	9	7	5	0.511
Unknown	1	0	2	0	1	1	
Hamilton Depression scale score, Month 12- Month 0	−5 (2.6)	−3 (2.6)	−2 (1.8)	−4 (2.5)	−3 (2.7)	−4 (2.9)	0.322
Unknown	1	0	2	1	0	1	

1 n; Mean (SD).

2 Fisher’s exact test; Kruskal-Wallis rank sum test.

**TABLE 3:** Comparison of change in functional status outcomes at Month 12 from baseline.

Treatment arm	Unadjusted beta estimate	95 % Confidence interval	p-value	Adjusted beta estimate^#^	95 % Confidence interval	p-value
Aspirin 0 + Atorvastatin 0	ref			ref		
Aspirin 75 mg + Atorvastatin 0	−0.40	−1.20, 0.43	0.337	−0.40	−1.2, 0.44	0.343
Aspirin 0 + Atorvastatin 20mg	−0.04	−0.90, 0.81	0.917	−0.04	−0.90,0.81	0.917
Aspirin 0 + Atorvastatin 80mg	−0.20	−1.00, 0.63	0.630	−0.20	−1.00, 0.64	0.632
Aspirin 75 mg + Atorvastatin 20mg	−0.24	−1.00, 0.57	0.561	−0.24	−1.10, 0.58	0.564
Aspirin 75 mg + Atorvastatin 80mg	−0.50	−1.30, 0.33	0.231	−0.50	−1.30, 0.34	0.236

# adjusted for baseline modified rankin score.

**TABLE 4:** Comparison of change in cognitive performance outcomes at Month 12 from month 3 scores.

Treatment arm	Unadjusted beta estimate	95 % Confidence interval	p-value	Adjusted beta estimate	95 % Confidence interval	p-value
Aspirin 0 + Atorvastatin 0	ref			ref		
Aspirin 75 mg + Atorvastatin 0	4.30	−0.48, 9.00	0.077	1.00	−2.10, 4.10	0.513
Aspirin 0 + Atorvastatin 20mg	1.50	−3.50, 6.60	0.545	−0.61	−3.80, 2.60	0.700
Aspirin 0 + Atorvastatin 80mg	1.00	−3.90, 5.90	0.683	−1.10	−4.20, 2.00	0.483
Aspirin 75 mg + Atorvastatin 20mg	−0.86	−5.50, 3.80	0.713	−2.60	−5.50, 0.36	0.084
Aspirin 75 mg + Atorvastatin 80mg	2.70	−2.10, 7.40	0.264	−0.14	−3.20, 2.90	0.925

# adjusted for baseline Montreal cognitive assessment score.
